# Missed nursing care in newborn units: a cross-sectional direct observational study

**DOI:** 10.1136/bmjqs-2019-009363

**Published:** 2019-06-06

**Authors:** David Gathara, George Serem, Georgina A V Murphy, Alfred Obengo, Edna Tallam, Debra Jackson, Sharon Brownie, Mike English

**Affiliations:** 1 Public Health Research, Kenya Medical Research Institute–Wellcome Trust Research Programme, Nairobi, Kenya; 2 Nursing and Midwifery, Aga Khan University School of Nursing and Midwifery East Africa, Nairobi, Kenya; 3 Public Health Research, KEMRI/Wellcome Trust Research Programme, Nairobi, Kenya; 4 Global Health, Bill and Melinda Gates Foundation, Seattle, Washington, USA; 5 National Nurses Association of Kenya, Nairobi, Kenya; 6 Registration and Licensing, Nursing Council of Kenya, Nairobi, Kenya; 7 Nursing and Midwifery, University of Technology Sydney, Sydney, New South Wales, Australia; 8 School of Medicine, Griffith University Faculty of Health, Gold Coast, Queensland, Australia; 9 Department of Paediatrics, University of Oxford, Oxford, UK

**Keywords:** health services research, nurses, patient safety, quality measurement, standards of care

## Abstract

**Background:**

Improved hospital care is needed to reduce newborn mortality in low/middle-income countries (LMIC). Nurses are essential to the delivery of safe and effective care, but nurse shortages and high patient workloads may result in missed care. We aimed to examine nursing care delivered to sick newborns and identify missed care using direct observational methods.

**Methods:**

A cross-sectional study using direct-observational methods for 216 newborns admitted in six health facilities in Nairobi, Kenya, was used to determine which tasks were completed. We report the frequency of tasks done and develop a nursing care index (NCI), an unweighted summary score of nursing tasks done for each baby, to explore how task completion is related to organisational and newborn characteristics.

**Results:**

Nursing tasks most commonly completed were handing over between shifts (97%), checking and where necessary changing diapers (96%). Tasks with lowest completion rates included nursing review of newborns (38%) and assessment of babies on phototherapy (15%). Overall the mean NCI was 60% (95% CI 58% to 62%), at least 80% of tasks were completed for only 14% of babies. Private sector facilities had a median ratio of babies to nurses of 3, with a maximum of 7 babies per nurse. In the public sector, the median ratio was 19 babies and a maximum exceeding 25 babies per nurse. In exploratory multivariable analyses, ratios of ≥12 babies per nurse were associated with a 24-point reduction in the mean NCI compared with ratios of ≤3 babies per nurse.

**Conclusion:**

A significant proportion of nursing care is missed with potentially serious effects on patient safety and outcomes in this LMIC setting. Given that nurses caring for fewer babies on average performed more of the expected tasks, addressing nursing is key to ensuring delivery of essential aspects of care as part of improving quality and safety.

## Introduction

Although progress has been made globally in reducing under-five mortality deaths in the first 28 days of life (the neonatal period) declined at a slower rate, particularly in sub-Saharan Africa.^1 2^ As a consequence, neonatal mortality contributes about 45% of mortality for children under 5 years.^3^ A recent review by Bhutta and colleagues indicated that high-impact low-cost interventions could avert more than 71% of neonatal deaths with 82% of this effect being attributable to facility-based care.^4^ However, quality of care for newborns in health facilities has been reported as poor in low/middle-income countries (LMIC).^5 6^ Most of these LMIC studies have focused on resource availability and processes of medical care with little detailed information on the quality and nature of care provided to sick newborns by nurses.

LMICs, especially sub-Saharan Africa, are also facing critical health workforce shortages with the global shortage estimated at over 7 million.^7^ In Kenya, Wakaba and colleagues reported that public sector nursing densities ranged from 0.008 to 1.2 per 1000 population across counties^8^ compared with an internationally suggested minimum health workforce threshold of 4.5/1000 population for doctors, nurses and midwives to achieve the Sustainable Development Goals.^9^ Nurses in hospitals are vested with the responsibility of delivering interventions prescribed by other providers (doctors, nutritionists, and so on) in addition to providing nurse-initiated interventions.^10^ In higher income countries there is a growing body of knowledge on the important contribution of nursing care in hospitals to patient safety,^11^ outcomes and care quality,^12^ with an association between nursing shortages and care being delayed or omitted.^13^ This latter phenomenon has been described as ‘implicit rationing’, ‘missed care’, ‘unmet nursing care needs’, ‘care left undone’ or ‘task incompletion’.^14^ Hereafter, we use the term missed care to encompass all these terms. These prior reports on missed care are based on nurse surveys, only two have focused on newborn care provision but within neonatal intensive care and they illustrate basic nursing care was missed with unexpected rise in patient volume/acuity and interruptions to respond to emergencies as the most common reason for care being missed.^15 16^ Similar findings have been reported in the only study we identified from Africa with the main nursing tasks left undone being comfort/talking to patients, educating patients and family and developing/updating nursing care plans/pathways.^17^ Authors of a recent systematic review recommended that researchers need to develop objective observational methods for quantifying missed care to advance this field further.^14^

Our aims were therefore to explore the extent of nursing care delivered to sick newborns in hospitals in an LMIC, going beyond prior reports that have focused predominantly on medical aspects of care,^5 18 19^ and develop and use direct observational methods to identify and quantify the nature of missed care in this setting. In doing this we had a secondary objective to explore how nursing shortages may be directly impacting neonatal nursing care provision.

## Methods and analysis

This was a cross-sectional study using direct observational methods to describe the essential neonatal nursing care given to individual sick newborns in Nairobi, Kenya. The study protocol is described in detail elsewhere.^20^

### Establishing essential nursing care practices

In earlier work Kenyan nursing experts and policymakers developed draft minimum standards for neonatal nursing care with recommendations on which tasks should be done and their frequency over 24-hour periods (see online supplementary table 1).^21^ Although these standards were initially developed by a small group of stakeholders (n=12), they have since been presented to wider nurse expert stakeholder groups and representatives from Ministry of Health, training institutions and development partners with interests in newborn health (Unicef, WHO) for validation and were considered acceptable standards. These standards take account of three different levels of illness severity in hospitalised newborns with categories A: the most acutely ill; B the moderately ill; and C the least ill. The nursing experts further agreed by consensus that if a baby receives 80% or more of recommended nursing care this would comprise a minimum threshold for adequate nursing care delivered.^21^ Standards for provision of nursing care have generally been neglected and these are to our knowledge the first explicitly developed for hospital care in Kenya. While these guidelines were developed for the Kenyan context the absence of reports in the literature of standards developed for similar settings suggests they may have wider value as has been the case for clinical guidelines.^22^

10.1136/bmjqs-2019-009363.supp2 Supplementary data

### Study sites and data collection

This study drew on earlier work that identified the facilities (n=31) providing inpatient newborn care for 24 hours, 7 days a week (hereafter referred to as 24/7) to the population of Nairobi.^23 24^ Among these hospitals 13/31 had more than 100 neonatal admissions annually and they provided care to over 96% of the entire sick-newborn population accessing care within Nairobi County. These 13 facilities were considered eligible for our study. We stratified these by workload (newborn admissions per year ≤500 low; >500 high) and sector (the public, private not for profit, hereafter referred to as mission hospitals, and private for profit, hereafter referred to as private hospitals). We purposively selected six hospitals, two from each sector, ensuring one high and one low workload facility in each sector. Purposive selection of hospitals was used as part of our aim was to span each sector to maximise variation in nurse to baby ratios and, because the proposed work was deemed potentially sensitive, we required strong support of the hospital administration. We used findings from a previous study that explored the readiness of hospitals (their organisation and resources) to provide a ‘structural quality score’ for each facility to help characterise the six selected facilities.^24^

### Study population and sampling strategy

All newborns admitted within the newborn unit in the six selected health facilities over the specific study period formed the potential study population. However, newborns who were at high risk of death within 12 hours, as defined by the clinician in charge (extremely low birthweight babies, babies requiring frequent resuscitation), needing specialised care/treatment (eg, scheduled for surgery, requiring transfer for ventilation or with gross congenital malformations) were deemed ineligible for ethical reasons and as the draft minimum standards were not applicable. Newborns whose guardian or nurse declined consent were excluded from the study.

Nurse staffing and routine activities may vary between weekdays and weekends and night and day. Care within newborn units is also often organised so that babies with different levels of disease severity are in different ward sections/rooms.^21^ In each hospital, a random sample of 12 shifts/time blocks of 12 hours (144 observation hours per hospital) were selected from within a 3-week period. We used stratified random sampling to ensure we observed three weekday day shifts, three weekday night shifts, three weekend day shifts and three weekend night shifts. Pilot data collection exercises confirmed it was logistically feasible for one observer to make direct observations of three babies located in adjacent cots in the same ward area for these 12 hours’ time blocks. Since care within the newborn units is typically organised so that babies with similar disease severity (categories A, B, C) are colocated in the same ward area, we therefore used purposeful sampling to ensure that for each shift group (eg, the three weekday day shifts) one focused on observing category A babies, one focused on category B babies and one focused on category C babies with three babies who met the inclusion criteria purposefully identified at the start of the 12 hours’ time block for this purpose (online supplementary figure 1). The 12-hour periods were selected because they span nursing shift change-overs and allowed observation of care round the clock. Detailed sampling and study procedures are provided in detail elsewhere.^20^

10.1136/bmjqs-2019-009363.supp1 Supplementary data

### Data collection

Data were collected between 1 September 2017 and 30 May 2018. We documented how often certain nursing tasks (listed in table 3) were undertaken in a 12-hour shift (07:00−19:00 or 19:00−07:00) using an observation checklist. The observers spent 1 week in the hospital before the 3-week period during which 12-hour shifts were randomly selected for observation. The familiarisation period enabled observers to learn the hospital environment and routines, introduce the study and gain consent from nurses. This 1-week familiarisation period also allowed the staff to become familiar with the observers aimed at reducing nurses’ efforts to modify their behaviour (the Hawthorne effect). Team or task nursing was the commonly used approach rather than primary nursing in provision of nursing to newborns. Therefore, over the 12 hours’ observation period, the care provided to three babies was typically provided by multiple nurses. As such, the baby to nurse ratio over a 12-hour shift was computed by dividing the total number of babies admitted in the unit with the number of nurses working during the shift. For instance, if there were 30 babies in the newborn unit and three nurses were providing care during a 12-hour shift, the resulting baby to nurse ratio was 10 babies to 1 nurse. Majority of the nurses practising within newborn units are registered general nurses trained at a diploma level (registered nurses) with no specialist training in newborn care. Within the study hospitals, we did not observe significant variation in the process of allocation of qualified nurses to different levels of acuity based on training or years of experience.

For each newborn selected for direct observation, the medical records were first reviewed and data on the diagnosis, disease severity and any specific interventions (eg, requirements such as phototherapy or oxygen) were collected. This initial information allowed the observer to determine the nature and number of expected nursing tasks to be delivered for each baby based on their illness severity (category A, B or C), the interventions they were receiving and the nursing care standards. We categorised tasks as nursing/clinical tasks that require physical interaction with the baby or mother/family member (for instance, feeding the baby, taking vital signs or providing counselling) or documentation tasks (eg, recording of vital signs) for which the observer checked nursing and medical records. Tasks are listed in table 3 and the observer recorded if a task was done or not done by a nurse (scored 0/1).

Observations were stopped if a baby was discharged, transferred out of a section or changed condition and became critically ill (when the minimum draft nursing standards did not apply). However, the data collected up to the point of exit were used to readjust denominators (see below). Similarly, if a baby’s care changed but they remained in the same observation area, this change was documented and the expected number of tasks revised. At the end of each 12-hour shift nursing and medical records were reviewed for evidence of documentation tasks.

Observations were made by a nutritionist, considered an appropriate cadre because they are familiar with the hospital environment, equipment, care processes and medical language, and would be considered a professional rather than an ‘outsider’. Moreover, we felt observing sick babies might be less distressing for a person with a health professional background. Using an observer who was not a nurse or clinician we felt might help overcome bias introduced by the observer relating their observations to their own standards of practice or being influenced by shared professional allegiances.

### Sample size and analysis

Our primary objective was to assess and quantify nursing care delivered to sick newborns and identify missed care. As such, we based our sample size estimations on the precision around proportions for individual tasks reported as done (or not done). We estimated that observing 216 babies (36 per hospital for 12 hours) would provide denominators of 108, 216 and 432 for the total number of times a task should be done (observed) assuming the task was required for all babies and standards indicated the task should be done once, twice and four times per 24 hours, respectively. Assuming a design effect of 2 to adjust for clustering of observed tasks within hospitals would allow us to report precision (95% CIs) around a statistically conservative proportion of 50% of expected tasks done of ±13.4%, 9.4% and 6.7%, respectively. The actual denominator for some tasks would, however, depend on the patterns of use of specific interventions (eg, phototherapy, and see table 3) reducing our reported precision. In the specific case of feeding, babies were often observed to have more than one type/route of feeding as an option. In such cases, we pooled data from different types of feeding (nasal gastric tube feeding, cup and spoon and breast feeding) so that a baby was documented as fed if they were observed to receive feeds using one or more of the above routes at the expected frequency.

For our primary objective, we pool our data across all babies observed and report as a proportion (with corresponding 95% CIs adjusted for clustering at the hospital level) the number of times a specific task was observed as done divided by the number of times it was expected to be done. Some tasks (eg, vital signs monitoring) should be done on all babies irrespective of the severity of illness/severity category and so the proportions reported represent aggregate measures across all babies and severity categories (table 3). Other tasks (eg, intravenous fluid or oxygen monitoring) might only be required in babies in severity categories A and B. Proportions reported therefore reflect performance in such subgroups (with appropriate cluster-adjusted CIs).

**Figure 1:**
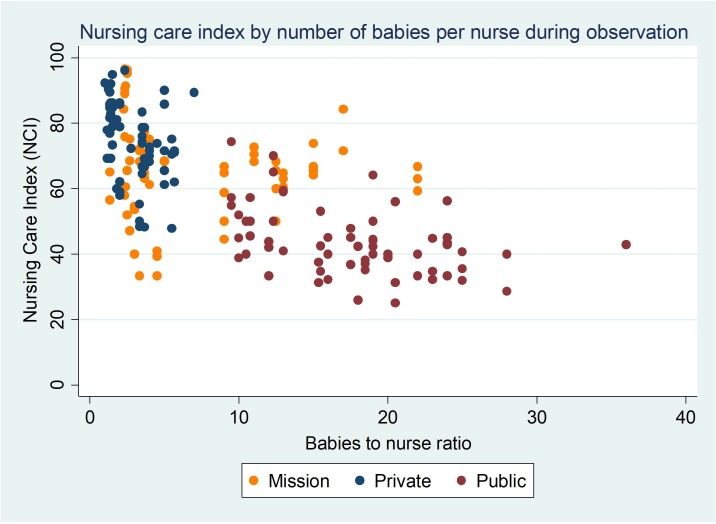
Nursing care index for each baby by number of babies per nurse across sectors.

In secondary analyses we created for each baby a denominator based on the total number of expected nursing tasks that should have been delivered based on the standards and the number of interventions each baby was receiving. This baby-specific denominator was then used to determine a proportion of expected tasks actually observed to be completed for each baby. This created a summary unweighted performance measure (all tasks given equal weight), the nursing care index (NCI), at individual level for which the denominator varies by diagnosis and case severity. As indicated above during the development of the minimum standards, local experts agreed that babies receiving 80% or more of their expected care tasks met a minimum threshold for adequate nursing care delivered.^21^ We therefore created a binary variable representing adequate nursing care delivered based on whether babies’ NCI was 80% or more and report the proportion of babies receiving adequate nursing care delivered. In further analyses we use the NCI to explore associations between this summary measure of care delivered at the baby level with characteristics of the hospital (sector), of the shift (the baby to nurse ratio, categorised into <3 babies; 4–11 babies and >12 babies per nurse) and of the baby (postnatal age categorised into ≤3 days; 3–7 days and 8–28 days, birth weight categorised into ≤1499 g; 1500–1999 g; 2000–2499 g; and ≥2500 g and severity category). To define the baby to nurse ratio categories, the distribution of data on baby to nurse ratio was used to ensure a reasonable number of observations in each category. Linear regression was used to explore associations between the NCI (dependent variable) and these hospitals, shift and baby characteristics in unadjusted models. Multivariable models were built to explore associations further using a stepwise forward selection procedure. Babies per nurse was included a priori as an independent covariable in preference to hospital identity with which it is strongly associated in our data set. We therefore could not include hospital identity in the regression models. We opted to use baby to nurse ratio, while acknowledging that this is also a proxy for sector (see figure 1) in our data set, as staffing ratios are a key parameter tracked and reported in most missed care literature. To build our multivariable model we used the Hosmer-Lemeshow criterion of a likelihood ratio test (LRT) with p<0.2 in the univariable analysis to identify possible covariable of interest. We added covariables starting with those with the strongest association in univariable analyses. LRTs (p<0.05) were used to determine whether additional factors added to the model should be retained in a final model. In a linked exercise, the LRT was also used to examine whether babies per nurse be included as a continuous or categorical variable. All analyses were conducted using the statistical analysis software STATA V.13.

Written informed consent was sought from both mothers and nurses while hospital management teams provided permission to conduct the study in the hospitals.

## Results

Data were collected from six hospitals spanning public, private and mission sectors. Of the 13 hospitals that met our inclusion criteria as possible study hospitals, we identified six hospitals to be included in the study. One medium-sized private hospital (657 annual admissions) declined to participate in the study citing hospital policy on access of medical records and patient privacy, a replacement hospital with similar characteristics was identified from the remaining seven hospitals. No refusals from families/caregivers were reported. The annual neonatal workload for these hospitals ranged from 123 to 1438 newborns admitted per year while the annual total deliveries ranged from 1398 to 6620 births. In a previous study, we assessed the availability of basic infrastructural resources for providing care (structure index) in accordance with Kenyan guidelines.^24^ The availability of basic infrastructural resources was considered at least good (>80%) in all six hospitals and varied from 81% to 92%. The two mission hospitals were heterogeneous, one was more similar to a private hospital while the other had staffing ratios and workloads similar to those in public hospitals. A summary of hospital characteristics is presented in table 1.

**Table 1:** Hospital characteristics by workload and availability of resources to provide care (structure index)

Hospital	Sector	Cots	Annual newborn admissions	Annual total deliveries	Structure index (score 0–100)*	Mortality by sector†
Hospital 1	Mission	8	1438	6620	91	5.9
Hospital 2	Mission	15	160	1305	87	5.9
Hospital 3	Private	30	1816	2273	92	7.3
Hospital 4	Private	25	123	1398	91	7.3
Hospital 5	Public	21	1006	5457	81	16.5
Hospital 6	Public	15	299	6180	90	16.5

*Structure index comprised items from the following domains: infrastructure, laboratory services, hygiene equipment, safe delivery equipment and drugs for mothers, resuscitation equipment for newborns in the delivery ward, essential equipment in the newborn unit, intravenous fluids and feeds in the newborn unit and essential drugs in the newborn unit.^24^

†Crude inpatient neonatal mortality data estimates are based on study where 21% of the outcome data were missing and are therefore likely to be biased.^41^

A total of 216 babies were observed (described in table 2 and online supplementary table 2 for hospital-specific results) against a direct observation checklist with an equal number of babies (72) in each sector. The majority of the babies were aged less than 7 days, 61% (129), while 33% (70) and 59% (126) weighed <1500 g and were born via caesarean, respectively. Of those delivered via caesarean section, 42% (53/126) were from the private sector. The primary reasons for admission were prematurity/low birth weight 43% (92), respiratory distress syndrome 19% (42) and severe jaundice 11% (24). There were relatively equal numbers of observations across the sample stratifying variables (sector, neonatal care category and nursing shift). A baby was only observed for one 12-hour shift and not in any subsequent periods.

10.1136/bmjqs-2019-009363.supp3 Supplementary data

**Table 2:** Characteristics of babies observed

Characteristic	n (%) n=216
Shift of observation	
Weekday day	59 (27.3)
Weekday night	54 (25.0)
Weekend day	50 (23.1)
Weekend night	53 (24.5)
Neonatal care category	
A (severe illness)	69 (31.9)
B (moderate severity)	75 (34.7)
C (stable)	72 (33.3)
Gender	
Male	122 (56.7)
Female	93 (43.3)
Age categories (days)	
≤2	49 (23.0)
3–7	80 (37.6)
8–28	84 (39.4)
Pooled birth weight categories (kg)	
<1.4	70 (32.6)
1.5 to <1.9	50 (23.3)
2.0 to <2.4	22 (10.2)
≥2.5	73 (34.0)
Baby:nurse ratio	
1–3 babies/nurse	84 (39.1)
4–11 babies/nurse	50 (23.3)
≥12 babies/nurse	81 (37.7)
Type of delivery	
Spontaneous vaginal delivery	81 (37.9)
Caesarean section	126 (58.9)
Assisted vaginal delivery	7 (3.3)
Primary diagnosis at observation	
Premature, LBW	92 (42.6)
Respiratory distress syndrome	42 (19.4)
Jaundice	24 (11.1)
Birth asphyxia	17 (7.9)
Neonatal sepsis	9 (4.2)
Meconium aspiration	7 (3.2)
Hypoxic ischaemic encephalopathy	5 (2.3)
For observation/accommodation	8 (3.7)
Other	12 (5.6)

LBW, low birth weight.

In table 3, we present the proportions when specific expected tasks were observed to be completed by nurses using data pooled across all babies observed. The tasks most commonly completed by nurses were nursing care handing over for babies between shifts (97%), checking and, where necessary, changing diapers (96%), checking eyes for damage from phototherapy, turning of babies on phototherapy (91%) and supporting mothers practising kangaroo mother care (91%). The least done tasks included nursing review of newborns (38%), cord care (38%), turning/repositioning (38%), cleaning eyes and checking for discharge/infection for babies on phototherapy (38%), oxygen saturation monitoring (34%) and skin assessment for babies on phototherapy (15%). Of the vital signs, oxygen saturation (required six hourly for babies on oxygen or in category A or B) was the least done, 34% (49/144), but pulse, respiratory rate and temperature monitoring (required for category A, B and C babies) were also done on fewer than 60% of occasions. For documentation tasks, treatment and fluid administration were the most documented, 97% and 91% of the episodes, respectively, while the least documented tasks were turning (27%) and communication with the parent (25%). Online supplementary table 3 describes in detail the number of expected tasks as per neonatal nursing guidelines and the proportion of these tasks completed by neonatal care categories and hospital sector. The median number of expected tasks (effective denominator) per baby was 23 (IQR 20–28, minimum and maximum 16 and 44, respectively). For all 216 babies observed, the mean NCI was 60% (95% CI 58% to 62%; range 24–96) (table 4). Variations in the NCI became apparent when observations were stratified by the sector and day/time of observation as well as by baby-specific characteristics (eg, clinical category) and by the baby to nurse ratio on the whole ward at the time of the observations. For example, higher proportions of care were done in the private sector (mean 74%; 95% CI 71% to 77%), in the 1–3 babies per nurse category (mean 73%; 95% CI 70% to 7%), and among sicker babies—category A (mean 63%; 95% CI 59% to 68%). A generally similar pattern was observed if nursing/clinical tasks and documentation tasks were considered separately with a suggestion that more documentation tasks were done for category A babies (mean documentation-specific NCI 68%; 95% CI 62% to 73%) compared with category C babies (mean documentation-specific NCI 50%; 95% CI 45% to 56%). To explore the proportion of babies receiving a minimum threshold of adequate nursing care delivered, we applied our previously defined cut-off of ≥80% of the required nursing tasks per baby being done. Overall, 14% (95% CI 10% to 20%) of the babies received a minimum threshold of adequate nursing tasks done by this criterion. While none of the babies in the public sector met this criterion, 31% (22) and 13% (9) of the babies in the private and mission sectors achieved this threshold, respectively. Although suboptimal, higher proportions of babies who were sicker (category A, 23% (16)) and where staffing ratios were 1–3 babies per nurse (32% (27)) were observed to receive minimum threshold of adequate nursing care (table 4).

10.1136/bmjqs-2019-009363.supp4 Supplementary data

**Table 3:** The number of expected tasks as per neonatal nursing guidelines and the proportion of these tasks completed by domain and type

Domain	Task type	Task	Required for all babies observed in a 12-hour shift	Frequency in 24 hours according to standards*	Expected tasks assuming 12 hours’ observation shifts and adjusting for category/interventions (n)	Tasks done, n (%)	95% CI
Nursing/clinical tasks	General nursing	Handing over nursing care between shifts	Yes	2	216	210 (97.2)	87 to 99
		Nursing review of newborns	Yes	2	216	83 (38.4)	16 to 67
		Baby cleaned	No	1	126	83 (65.9)	43 to 83
		Linen changed	No	1	126	70 (55.6)	26 to 81
		Nurse attends ward round†	No	1	75	64 (85.3)	21 to 99
		Checking and changing diaper as required	Yes	8	216	207 (95.8)	87 to 99
		Communication to parent	Yes	1	216	105 (48.6)	30 to 67
		Handwashing/scrub‡	Yes	2	216	200 (92.6)	67 to 99
		Cord care where required	No	1	110	42 (38.2)	17 to 65
		Temperature monitored§	Yes	4	216	127 (58.8)	20 to 89
		Respiration monitored§	Yes	4	216	107 (49.5)	16 to 83
		Pulse monitored§	Yes	4	216	122 (56.5)	19 to 88
		Oxygen saturation monitored§	No	4	144	49 (34.0)	9 to 72
		Turning done as required	Yes	8	216	81 (37.5)	13 to 71
		Feeding three hourly as required	No	8	180	126 (70.0)	58 to 80
	Phototherapy care	Clean eyes and check for discharge/infection	No	4	34	12 (35.3)	11 to 71
		Eye pad changed	No	2	34	12 (35.3)	12 to 69
		Skin assessment¶	No	4			
		Skin assessment 1			34	19 (55.9)	27 to 81
		Skin assessment 2			34	5 (14.7)	1 to 68
		Check eyes for damage from phototherapy¶	No	4			
		Check eyes for damage 1			34	31 (91.2)	53 to 99
		Check eyes for damage 2			34	19 (55.9)	26 to 82
		Turning/positioning done¶	No	6			
		Turning/positioning done 1			34	31 (91.2)	48 to 99
		Turning/positioning done 2			34	26 (76.5)	48 to 92
		Turning/positioning done 3			32	14 (43.8)	23 to 66
	Oxygen therapy care	Oxygen regulated			76	61 (80.3)	36 to 97
		Check nostril tube position¶	No	8			
		Check nostril tube position 1			75	61 (81.3)	60 to 93
		Check nostril tube position 2			76	42 (55.3)	17 to 88
		Check nostril tube position 3			76	42 (55.3)	19 to 87
		Check nostril tube position 4			76	36 (47.4)	13 to 84
	Intravenous fluids	Fluids regulated as required**	No	2	21	16 (76.2)	11 to 99
	Intravenous treatment	Cannula flushed before giving intravenous treatment††	No	2	126	51 (40.5)	7 to 86
	KMC	Counselling and supporting mother to initiate and continue with KMC	No	2	32	29 (90.6)	57 to 99
		Supervision of the mother for correct KMC practice	No	2	32	24 (75.0)	31 to 95
Documentation tasks	Documentation	Clinical nursing review	Yes	2	216	107 (49.5)	22 to 77
		Planned care	Yes	2	216	140 (64.8)	20 to 93
		Vital signs	Yes	2	216	154 (71.3)	22 to 96
		Treatment documented	No	2	150	146 (97.3)	90 to 99
		Ward round recommendations	No	1	75	55 (73.3)	44 to 91
		Phototherapy documentation	No	2	31	19 (61.3)	18 to 92
		Summary of feeds intake	No	2	180	137 (76.1)	33 to 95
		Oxygen therapy	No	2	76	57 (75.0)	43 to 92
		Health talks/parent communications‡‡	Yes	2	216	53 (24.5)	6 to 63
		Charting of fluids administered	No	2	66	60 (90.9)	73 to 97
		Turning/positioning	Yes	2	216	59 (27.3)	6 to 67

*For instance, for tasks with a frequency of 2 in 24 hours we would observe one task in a 12-hour shift.

†Only one doctors ward round was expected in 24 hours.

‡At first contact with patient only since it was difficult to establish a denominator since handwashing should be done before each time the nurse makes contact with the patient.

§Monitoring done as per draft neonatal nursing guidelines.

¶Tasks have multiple sub-items.

**During the observation shift or when fluid was running, evidence for an attempt to regulate the rate.

††For twice daily medication, we would observe two tasks in 24 hours.

‡‡Health talks/parents are supposed to be continuous; however, we are interested in at least two sessions in 24 hours (one during the day and one during the night shift).

KMC, kangaroo mother care.

**Table 4:** Mean nursing care index and proportion of babies with adequate nursing care delivered

	Mean (SD) nursing care index			Proportion of babies with adequate nursing care delivered (NCI≥80%)
	Overall	Nursing/clinical tasks	Documentation tasks	n/N (%)
Shift of observation				
Weekday day	61.9 (57.4–66.3)	63.6 (59.4–67.9)	57.5 (49.9–65.2)	9/59 (15.3)
Weekday night	58.5 (53.6–63.4)	58.1 (52.4–63.9)	59.3 (54.4–64.2)	6/54 (11.1)
Weekend day	62.9 (58.1–67.7)	64.1 (58.9–69.4)	59.9 (53.9–65.9)	7/50 (14.0)
Weekend night	58.2 (52.9–63.5)	59.6 (54.0–65.3)	54.5 (48.2–60.7)	9/53 (17.0)
Neonatal care category				
A (severe illness)	63.3 (58.8–67.8)	61.2 (56.3–66.0)	67.8 (62.4–73.2)	16/69 (23.2)
B (moderate severity)	60.0 (55.8–64.3)	61.6 (56.8–66.3)	55.6 (50.6–60.7)	12/75 (16.0)
C (stable)	57.9 (54.2–61.7)	61.5 (57.6–65.3)	50.4 (45.1–55.6)	3/72 (4.2)
Baby:nurse ratio				
1–3 babies/nurse	72.9 (69.8–75.9)	73.7 (70.2–77.3)	71.3 (67.4–75.1)	27/84 (32.1)
4–11 babies/nurse	61.1 (57.3–64.9)	62.1 (58.1–66.1)	59.1 (52.8–65.3)	3/50 (6.0)
≥12 babies/nurse	47.0 (43.9–50.1)	48.4 (44.9–51.8)	43.0 (38.5–47.4)	1/81 (1.2)
Sector				
Mission	64.8 (61.5–68.0)	65.2 (61.6–68.9)	64.0 (59.7–68.2)	9/72 (12.5)
Private	73.9 (71.2–76.6)	74.6 (71.2–78.0)	72.7 (69.8–75.7)	22/72 (30.6)
Public	42.4 (40.0–44.8)	44.4 (41.3–47.4)	36.6 (31.8–41.5)	0/72 (0.0)

NCI, nursing care index.

### Hospital and baby characteristics associated with mean NCI

Initial univariable analyses suggested that a lower NCI was associated with a baby having a weight ≥1500 g, higher baby to nurse ratios on a shift (a 26-point reduction in mean NCI when there were ≥12 babies per nurse compared with 1–3 babies per nurse) and observations made in the public sector compared with the mission sector (22-point reduction in the mean NCI) (table 5). Meanwhile a higher NCI was associated with a postnatal age >8 days and care in the private sector. In the multivariable analysis that included baby to nurse ratio but excluded sector babies, age, neonatal care category and baby to nurse ratio were identified as associated with the NCI based on the LRT (p=0.005). In this multivariable model, a baby being in category C was associated with an 8-point reduction in mean NCI when compared with category A babies, and when there were ≥12 babies per nurse or 4–11 babies per nurse this was associated with a 24-point and 12-point reduction in NCI when compared with shifts when there were 1–3 babies per nurse. A postnatal age >8 days was associated with a 7-point higher NCI when compared with babies aged ≤2 days.

**Table 5:** Univariable and multivariable models for the association of mean NCI with baby and hospital characteristics

	Model 1: univariable associations				Model 2: multivariable associations		
	Coefficient	95% CI	P value	R^2^	Coefficient	95% CI	P value
Gender							
Male	Ref			0.003			
Female	1.99	−2.84 to 6.81	0.418				
Birth weight (kg)							
<1.4	Ref			0.026			
1.5 to <1.9	−7.75	−14.23 to −1.28	0.020				
2.0 to <2.4	−3.37	−11.92 to 5.17	0.440				
≥2.5	−3.84	−9.68 to 2.01	0.200				
Age (days)							
≤2	Ref			0.061	Ref		
3–7	−0.16	−6.34 to 6.02	0.959		1.78	−3.06 to 6.64	0.469
8–28	8.82	2.70 to 14.95	0.005		7.46	2.55 to 12.36	0.003
Nursing shift							
Day				0.013			
Night	−4.03	−8.79 to 0.73	0.100				
Neonatal care category							
A (severe illness)	Ref			0.015	Ref		
B (moderate severity)	−3.25	−9.09 to 2.59	0.274		−4.27	−8.78 to 0.23	0.063
C (stable)	−5.34	−11.23 to 0.56	0.076		−7.65	−12.29 to −3.02	0.001
Baby:nurse ratio							
1–3 babies/nurse	Ref			0.406	Ref		
4–11 babies/nurse	−11.79	−16.65 to −6.92	<0.001		−11.49	−16.26 to −6.73	<0.001
≥12 babies/nurse	−25.89	−30.13 to −21.65	<0.001		−24.41	−28.64 to −20.17	<0.001
Sector							
Mission	Ref			0.556			
Private	9.13	5.21 to 13.05	<0.001				
Public	−22.40	−26.32 to −18.49	<0.001				

NCI, nursing care index.

The strong apparent relationship between NCI measured for each baby and the baby to nurse ratio of the shift being observed was further explored in a simple scatter plot (figure 1). This demonstrates the strong relationship between sector and baby to nurse ratio and thus the relationship between sector and NCI apparent in univariable analysis. In the private sector the median ratio was 3 babies to 1 nurse with a maximum ratio of 7 babies to 1 nurse. In the public sector the median ratio was 19 babies to 1 nurse with a minimum of 10 and a maximum exceeding 25 babies per nurse.

## Discussion

The aim of this study was to quantify nursing care tasks that can be observed that were delivered to sick newborns and identify missed care (tasks done or left undone) within a set of Kenyan newborn units. Task completion varied greatly overall and across hospital sector and newborn illness severity category. We observed omission of nursing tasks that might directly influence the baby’s outcome, for instance, feeding, monitoring of vital signs and appropriate use of interventions like fluids and oxygen. This highlights potentially critical safety issues, although our study was not designed to explore the effects on patient outcomes. These specifically missed tasks are likely to be compounded by indirect effects of missed care linked to poor communication between nurses and patients and among teams of carers.^25^ Communication with and education of mothers or caregivers, such as explaining the baby’s illness and management and teaching them how to safely feed their baby, was provided on less than half the occasions expected. These aspects of missed care may adversely affect mothers’ experience of care and influence babies’ early recovery and longer term maternal-neonatal bonding.^25 26^ Interprofessional and intraprofessional communication is likely undermined by, for example, poor documentation and inability of nurses to engage in medical rounds. Both may adversely affect the teamwork that is critical to providing safe, effective care in high-pressure clinical environments.^27 28^

In our secondary analysis we developed a measure that aggregated all the (observable) tasks done per baby, the NCI. The mean proportion of expected tasks done per baby was 60% overall. The threshold recommended by local experts representing minimum threshold of adequate nursing care delivered was rarely achieved (14% babies). The NCI varied in association with sector being highest in the private sector. However, there was a strong association between sector and the number of babies that each nurse was caring for. No babies were observed in the private sector when there were >7 babies per nurse while no babies in the public sector were observed when there were <10 babies per nurse. Failure to take account of this dramatically different nursing workload could, mistakenly we believe, be interpreted as suggesting that nurses generally perform better in the private sector. Focusing on the number of babies per nurse, our findings suggest this strongly related to the proportion of tasks completed (NCI). Our model suggests a 24% reduction in the NCI when there was 1 nurse per 12 or more babies compared with 1 nurse to up to 3 babies. We believe that the relationship between staffing levels and care received also mediates the apparent effect of shift timing on NCI (with care at night scoring lower than in the days). The obvious implication is that to improve quality of care, it is imperative that workforce deficits are addressed. These findings contribute to the growing body of evidence linking inadequate staffing and missed care. Studies undertaken in Sweden,^29^ across Europe^30^ and in England^13^ have reported associations between staffing and nursing care left undone. Additionally, the number of patients per nurse and the number of nursing care hours per patient-day have been associated with missed care.^31 32^ However, most of this literature is based on data from nurse surveys of self-reported missed care and are from high-income country (HIC) settings. While improving nurse numbers is key, our data illustrate considerable variation in the NCI with the same nurse to baby ratios (figure 1). This suggests there is also some potential for improving care by learning what steps nurses take in some settings to achieve high performance despite significant challenges through efforts to study ‘positive deviants’.

Additional findings from our secondary analysis suggest that babies who were more severely ill (category A) received higher levels of nursing care (8% higher NCI) compared with stable babies (category C) in the adjusted multivariable analysis. We hypothesise that nurses may feel stable babies are out of danger and hence prioritise care provision to babies who are perceived to be at higher risk of death. These findings are consistent with parallel ethnographic work conducted by our team that suggests nurses have to engage in ‘sub-conscious triage’ when the volume of work is overwhelming^33^ as well as with wider literature reporting that nurses often prioritise medical or technical interventions at the expense of social and relational aspects of care.^25^ New technologies are widely felt to offer great promise for improving newborn outcomes but are most likely to be used in the sickest babies. Their introduction may further increase time spent on this group to the neglect of babies who are apparently less ill, potentially putting this group at risk of deterioration, or delay to their recovery. Moreover, these technologies still require human resources to support their use and could potentially exacerbate the general problem of missed care in settings with critical workforce deficits. While our data illustrate the extent of missed care taking the perspective of the baby, there are also likely to be important effects on nurses themselves of such high workloads and their own perception of failing to meet professional expectations of care. The exhaustion and burnout that are potential consequences are important detrimental effects on the emotional and psychological well-being of nurses^34^ and on sustainability of this crucial workforce.

The gaps in care we highlight underscore the urgent need for system strengthening to support the nursing workforce in LMIC and for quality improvement initiatives and research on service redesign to focus on nursing. As part of a wider programme of work, we observed that nurses’ time is often taken up by tasks that are not necessarily core to the nursing role. Examples include clerical tasks such as organising patient files, receiving telephone calls and billing, collecting supplies from stores and ward cleaning of baby cots and equipment. These non-patient-facing activities take up a significant amount of their time.^33^ Opportunities therefore exist to refocus nursing practice on skilled tasks for which they are specifically trained and reassign some tasks to other workers. Such approaches may enhance the professional status of nurses and make most efficient use of human resources through, for example, specific forms of task sharing. In HICs healthcare assistants support nursing care provision by undertaking non-technical tasks.^35 36^ In LMICs, including Kenya, task sharing/shifting from doctors to clinical officers (physician assistants) and nurses has been implemented to support care provision for HIV, tuberculosis and non-communicable diseases.^37 38^ However, delegation of some tasks to a supportive cadre needs careful consideration to ensure adequate supervision and patient safety.^39^ Furthermore, it should be clear that addressing the nursing workforce deficit is the first priority which may be complemented by introducing support workers.

Our results need to be interpreted in light of the following limitations. The use of direct observational methods limited the nursing tasks assessed to those that can be observed and we might have underestimated the magnitude of tasks done (or not done). Interestingly, we did note that care was sometimes documented as done when this was not corroborated by our observations, suggesting observations may be more accurate than record review. Observations might be influenced by observer bias and are at risk of Hawthorne effects. We developed through extensive piloting a highly structured checklist and provided careful training to help overcome these limitations in addition to a 1-week familiarisation period for observers in each hospital before the start of formal observations. We did not evaluate interobserver variability within the main study. A study team member and the four observers recruited did train together on the observational methods over a 1-week period during which we evaluated the observers’ performance against the study team member as the reference. Similar evaluations were conducted for 2 days in each hospital during the 1-week familiarisation period before start of the actual data collection. In these training exercises observers demonstrated >95% concordance with the observations of the study team member. During data collection there were weekly supervision visits to ensure consistency in data collection and adherence to study standard operating procedures.

We purposefully selected a relatively small sample of hospitals in one city that varied by sector and workload (annual admissions 106–1319) and excluded the sickest babies from our sample. This selection limits the generalisability of our findings although extremely sick newborns are a minority on the wards we studied. Despite the small number of hospitals studied, we feel the inclusion of different sectors with different organisational capacities provides useful insights on the nature and magnitude of missed care. The very different baby to nurse ratios found in the private and public sectors do, however, preclude our ability to explore any effect that the sector may have on our missed care measures and we make the assumption that it is baby to nurse ratio that is the major determinant of missed care. As a result, the findings of our exploratory analyses must be interpreted very cautiously although they are consistent with wider literature on the association between nurse staffing and missed care. The NCI we used may also be criticised for not taking account of the relative importance of some tasks (all are given equal weight). It does, however, have the advantage of being intuitive and easily understood and is based on tasks an expert panel proposed were all relevant to achieving a minimum standard of care while the allocation of task-specific weighting values could itself be very contested and has not to our knowledge been attempted in prior work on missed nursing care.

## Conclusion

Our work addresses an important gap in the global literature on quantifying the care delivered by nurses using direct observational methods. To the best of our knowledge, this is the first such study in a low-resource setting and it drew on development of local, contextually relevant standards. We observed great variation in task completion with potentially important implications for patient well-being and safety. Aggregating nursing tasks within babies, average task completion was 60%. Our exploratory analysis suggests a strong relationship between the high levels of missed care observed and the high baby to nurse ratios found especially in the public sector. Improving quality of care and its contribution to newborn survival clearly demands an expansion of the nursing workforce, potentially complemented by additional human resource innovations. Failure to address critical workforce issues will mean missed care remains common and undermine efforts to deliver high-impact, low-cost interventions for small and sick babies. While the focus of our work was newborn units in one city our wider experience suggests similar challenges are faced on paediatric and other hospital wards in Kenya and probably many other African settings. Our data therefore lend support to initiatives highlighting the critical role nurses play in care provision such as the ‘Nursing Now campaign,’ a global campaign aiming to improve health by raising the profile and status of nursing worldwide.^40^

## References

[1] Lawn JE, Blencowe H, Oza S, et al Every newborn: progress, priorities, and potential beyond survival. The Lancet 2014;384:189–205. 10.1016/S0140-6736(14)60496-7 24853593

[2] Requejo J, Cesar VBJ A decade of tracking progress for maternal, newborn and child survival, 2015. 10.1016/S0140-6736(15)00519-X PMC7613171 26477328

[3] World Health Organization : Countdown to 2015: a decade of tracking progress for maternal, newborn and child survival. Geneva: World Health Organization, 2015.

[4] Bhutta ZA, Das JK, Bahl R, et al Can available interventions end preventable deaths in mothers, newborn babies, and stillbirths, and at what cost? The Lancet 2014;384:347–70. 10.1016/S0140-6736(14)60792-3 24853604

[5] Vesel L, Manu A, Lohela TJ, et al Quality of newborn care: a health facility assessment in rural Ghana using survey, vignette and surveillance data. BMJ Open 2013;3 10.1136/bmjopen-2012-002326 PMC3651975 23667161

[6] Opondo C, Ntoburi S, Wagai J, et al Are hospitals prepared to support newborn survival? - an evaluation of eight first-referral level hospitals in Kenya*. Trop Med Int Heal 2009;14:1165–72. 10.1111/j.1365-3156.2009.02358.x PMC2751740 19695001

[7] Campbell J, Dussault G, Buchan J, et al A universal Truth: No Health without a Workforce In: Gene, 2013.

[8] Wakaba M, Mbindyo P, Ochieng J, et al The public sector nursing workforce in Kenya: a county-level analysis. Hum Resour Health 2014;12 10.1186/1478-4491-12-6 PMC3913960 24467776

[9] World Health Organization Health workforce requirements for universal health coverage and the sustainable development Goals. human resources for health observer, 2016

[10] All-Party Parliamentary Group on Global Health Triple impact: how developing nursing will improve health, promote gender equality and support economic growth, 2016.

[11] Aiken LH, Sermeus W, Van den Heede K, et al Patient safety, satisfaction, and quality of hospital care: cross sectional surveys of nurses and patients in 12 countries in Europe and the United States. BMJ 2012;344:e1717 10.1136/bmj.e1717 22434089 PMC3308724

[12] Kane RL, Shamliyan TA, Mueller C, et al The association of registered nurse staffing levels and patient outcomes: systematic review and meta-analysis. Med Care 2007;45:1195–204. 10.1097/MLR.0b013e3181468ca3 18007170

[13] Ball JE, Murrells T, Rafferty AM, et al ‘Care left undone’ during nursing shifts: associations with workload and perceived quality of care. BMJ Qual Saf 2014;23:116–25. 10.1136/bmjqs-2012-001767 PMC3913111 23898215

[14] Jones TL, Hamilton P, Murry N Unfinished nursing care, missed care, and implicitly rationed care: state of the science review. Int J Nurs Stud 2015;52:1121–37. 10.1016/j.ijnurstu.2015.02.012 25794946

[15] Lake ET, de Cordova PB, Barton S, et al Missed nursing care in pediatrics. Hosp Pediatr 2017;7:378–84. 10.1542/hpeds.2016-0141 28611146 PMC5485353

[16] Tubbs-Cooley HL, Pickler RH, Younger JB, et al A descriptive study of nurse-reported missed care in neonatal intensive care units. J Adv Nurs 2014:1–12. 25430513 10.1111/jan.12578

[17] Bekker M, Coetzee SK, Klopper HC, et al Non-nursing tasks, nursing tasks left undone and job satisfaction among professional nurses in South African hospitals. J Nurs Manag 2015;23:1115–25. 10.1111/jonm.12261 25345386

[18] Gathara D, Opiyo N, Wagai J, et al Quality of hospital care for sick newborns and severely malnourished children in Kenya: a two-year descriptive study in 8 hospitals. BMC Health Serv Res 2011;11 10.1186/1472-6963-11-307 PMC3236590 22078071

[19] Reyburn H, Mwakasungula E, Chonya S, et al Clinical assessment and treatment in paediatric wards in the north-east of the United Republic of Tanzania. Bull World Health Org 2008;86:123–39. 10.2471/BLT.07.041723 PMC2647389 18297168

[20] Gathara D, Serem G, Murphy GAV, et al Quantifying nursing care delivered in Kenyan newborn units: protocol for a cross-sectional direct observational study. BMJ Open 2018;8:e022020 10.1136/bmjopen-2018-022020 PMC6059345 30037876

[21] Murphy GAV, Omondi GB, Gathara D, et al Expectations for nursing care in newborn units in Kenya: moving from implicit to explicit standards. BMJ Glob Health 2018;3:e000645 10.1136/bmjgh-2017-000645 PMC5875677 29616146

[22] English M, Irimu G, Nyamai R, et al Developing guidelines in low-income and middle-income countries: lessons from Kenya. Arch Dis Child 2017;102:846–51. 10.1136/archdischild-2017-312629 28584069 PMC5564491

[23] Murphy GAV, Gathara D, Aluvaala J, et al Nairobi newborn study: a protocol for an observational study to estimate the gaps in provision and quality of inpatient newborn care in Nairobi City County, Kenya. BMJ Open 2016;6:e012448 10.1136/bmjopen-2016-012448 PMC5223685 28003285

[24] Murphy GAV, Gathara D, Abuya N, et al What capacity exists to provide essential inpatient care to small and sick newborns in a high mortality urban setting? - A cross-sectional study in Nairobi City County, Kenya. Plos One 2018;13:e0196585 10.1371/journal.pone.0196585 29702700 PMC5922525

[25] Papastavrou E, Andreou P, Efstathiou G Rationing of nursing care and nurse-patient outcomes: a systematic review of quantitative studies. Int J Health Plann Mgmt 2014;29:3–25. 10.1002/hpm.2160 23296644

[26] Lake ET, Germack HD, Viscardi MK Missed nursing care is linked to patient satisfaction: a cross-sectional study of US hospitals. BMJ Qual Saf 2016;25:535–43. 10.1136/bmjqs-2015-003961 PMC4794421 26376673

[27] Gonzalo JD, Kuperman E, Lehman E, et al Bedside interprofessional rounds: perceptions of benefits and barriers by internal medicine nursing staff, attending physicians, and housestaff physicians. J Hosp Med 2014;9:646–51. 10.1002/jhm.2245 25130404

[28] Gilson L, Barasa E, Nxumalo N, et al Everyday resilience in district health systems: emerging insights from the front lines in Kenya and South Africa. BMJ Glob Health 2017;2:e000224 10.1136/bmjgh-2016-000224 PMC5656138 29081995

[29] Ball JE, Griffiths P, Rafferty AM, et al A cross-sectional study of ‘care left undone’ on nursing shifts in hospitals. J Adv Nurs 2016;72:2086–97. 10.1111/jan.12976 27095463

[30] Ausserhofer D, Zander B, Busse R, et al Prevalence, patterns and predictors of nursing care left undone in European hospitals: results from the multicountry cross-sectional RN4CAST study. BMJ Qual Saf 2014;23:126–35. 10.1136/bmjqs-2013-002318 24214796

[31] Griffiths P, Recio-Saucedo A, Dall'Ora C, et al The association between nurse staffing and omissions in nursing care: a systematic review. J Adv Nurs 2018;74:1474–87. 10.1111/jan.13564 29517813 PMC6033178

[32] Aiken LH, Sloane DM, Bruyneel L, et al Nurse staffing and education and hospital mortality in nine European countries: a retrospective observational study. The Lancet 2014;383:1824–30. 10.1016/S0140-6736(13)62631-8 PMC4035380 24581683

[33] Nzinga J, McKnight J, Jepkosgei J, et al Exploring the space for task shifting to support nursing on neonatal wards in Kenyan public hospitals. Hum Resour Health 2019;17 10.1186/s12960-019-0352-x PMC6404312 30841900

[34] Khamisa N, Oldenburg B, Peltzer K, et al Work related stress, burnout, job satisfaction and general health of nurses. IJERPH 2015;12:652–66. 10.3390/ijerph120100652 25588157 PMC4306884

[35] Munn Z, Tufanaru C, Aromataris E Recognition of the health assistant as a delegated clinical role and their inclusion in models of care: a systematic review and meta-synthesis of qualitative evidence. Int J Evid Based Healthc 2013;11:3–19. 10.1111/j.1744-1609.2012.00304.x 23448325

[36] Twigg DE, Myers H, Duffield C, et al The impact of adding assistants in nursing to acute care hospital ward nurse staffing on adverse patient outcomes: an analysis of administrative health data. Int J Nurs Stud 2016;63:189–200. 10.1016/j.ijnurstu.2016.09.008 27653280

[37] Crowley T, Mayers P Trends in task shifting in HIV treatment in Africa: effectiveness, challenges and acceptability to the health professions. Afr J Prim Health Care Fam Med 2015;7. doi:10.4102/phcfm.v7i1.807. [Epub ahead of print: 30 Jul 2015]. PMC4564830 26245622

[38] Joshi R, Alim M, Kengne AP, et al Task shifting for non-communicable disease management in low and middle income countries – a systematic review. PLoS ONE 2014;9:e103754 10.1371/journal.pone.0103754 25121789 PMC4133198

[39] Butler M, Collins R, Drennan J, et al Hospital nurse staffing models and patient and staff-related outcomes. Cochrane Database Syst Rev 2011;(7):CD007019 10.1002/14651858.CD007019.pub2 21735407

[40] Nursing Now - raising the status of nursing worldwide. Available: https://www.nursingnow.org/ [Accessed 24 Dec 2018].

[41] Murphy GAV, Gathara D, Abuya N, et al What capacity exists to provide essential inpatient care to small and sick newborns in a high mortality urban setting? - A cross-sectional study in Nairobi City County, Kenya. PLoS One 2018;13:e0196585 10.1371/journal.pone.0196585 29702700 PMC5922525

