# Supplementary material for: Missed nursing care in newborn units: a cross-sectional direct observational study
Source: BMJ Qual Saf. 2019 Jun 6;29(1):19–30. doi: 10.1136/bmjqs-2019-009363 (PMC6923939; doi:10.1136/bmjqs-2019-009363)
Supplement: Supplementary data [file bmjqs-2019-009363supp002.pdf]

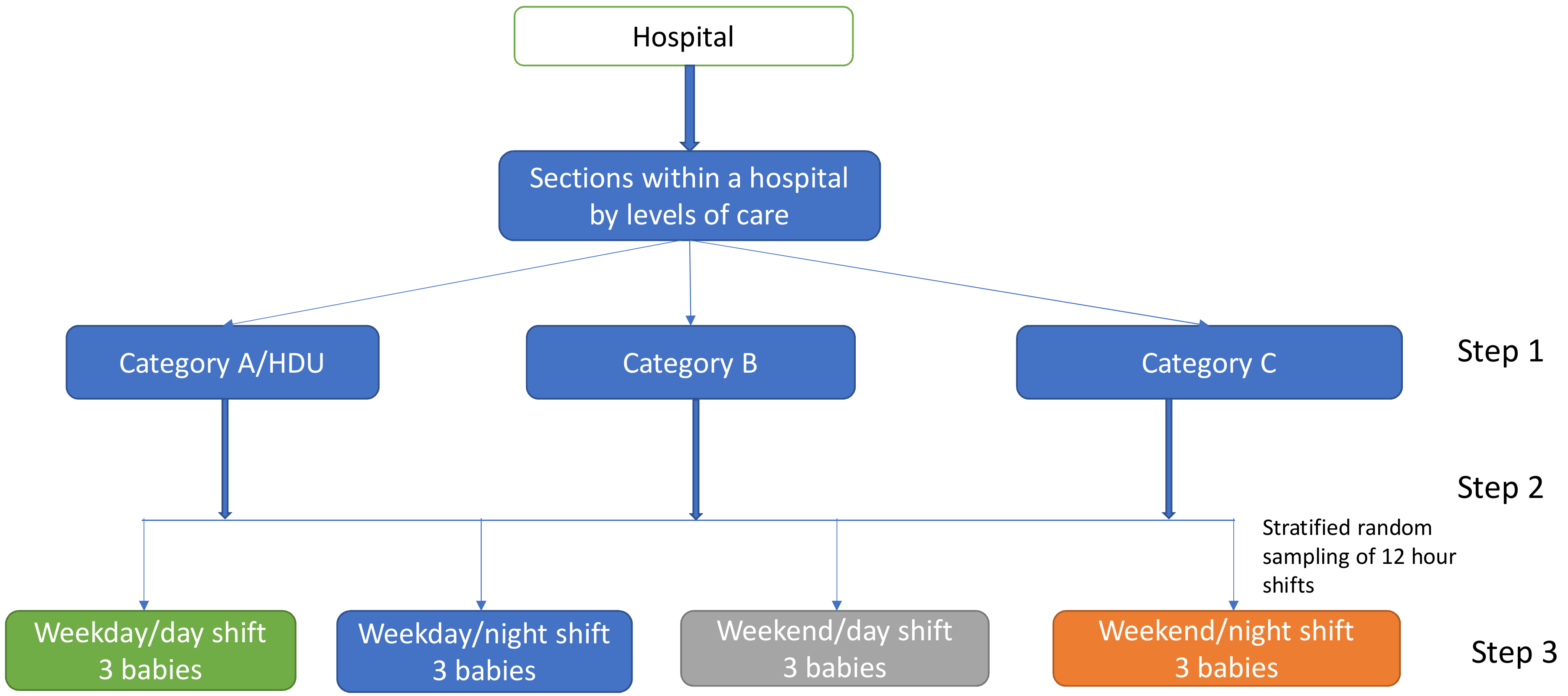

Supplementary figure 1: Steps for the sampling procedure

***Extracted from**: Gathara D, Serem G, Murphy GA V, et al. Quantifying nursing care delivered in Kenyan newborn units: protocol for a cross-sectional direct observational study. BMJ Open 2018;**8**:e022020. doi:10.1136/bmjopen-2018-022020*
