# Supplementary material for: Missed nursing care in newborn units: a cross-sectional direct observational study
Source: BMJ Qual Saf. 2019 Jun 6;29(1):19–30. doi: 10.1136/bmjqs-2019-009363 (PMC6923939; doi:10.1136/bmjqs-2019-009363)
Supplement: Supplementary data [file bmjqs-2019-009363supp001.pdf]

Supplementary table 1: Neonatal nursing care tasks recommendations on which tasks should be done and their frequency over 24 hour periods

Task Area	Task done by	Frequency (minimum daily requirement)	Comment
**Admission**			
Admission nursing history, clinical evaluation and vital signs	Nurse	On admission	
**Routine vital signs and monitoring**			
Temperature (including incubator temperature)	Nurse	*Normal:* 4 times daily/6 hourly<br>*Category C patients:* 2 times daily/12 hourly	KMC newborns: Vital signs monitoring and monitoring of general clinical condition
Pulse	Nurse		
Respiration	Nurse		
Checking and documenting oxygen saturation for babies not on oxygen (for babies on oxygen see section below).	Nurse	4 times daily/6 hourly	Although it is recognized that the availability of pulse oximeters is limited at present, checking oxygen saturation in sick babies not on oxygen should be promoted.
Skin colour	Nurse	*Normal:* 4 times daily/6 hourly<br>*Category C patients:* 2 times daily/12 hourly	Conduct together with vital signs monitoring
Jaundice	Nurse		
Respiratory effort	Nurse		
Abdominal distension	Nurse		
Weight	Nurse/clinician	Alternate days	
Input/output - general	Nurse	4 times daily/6 hourly	
Input - IV fluids	Nurse/clinician	Frequency depends on prescription. Infusion rate checked and documented 3 hourly.	Conduct together with vital signs
Input/output documentation (amount that has been infused)	Nurse	3 hourly	
**Regular patient checks/care**			
Changing diapers/checking for stool and urine	Nurse/patient's family	As required	Done with vital signs and document passing stool and urine during diaper change
Cleaning/bathing/clothing	Patient's family	As required	
Changing bed linens	Nurse/patient's family	As required	
Incubator monitoring and settings	Nurse	During shift changes/per shift	
Wound care (checking/renewing dressings)	Nurse	As required	
**Administering interventions/doing investigations**			
Taking venous blood	Nurse/clinician	As required	Nurses can perform this task if they have the skills to do so and if the clinicians charged with the responsibility are unavailable.
Taking heel-prick blood	Nurse/clinician	As required	
Collecting urine / stool	Nurse/patient's family	As required	
Resuscitation with bag valve mask	Multidisciplinary	As required	
**Drugs and vaccines**			
Drug preparation	Nurse	As per drug schedule	
Dilutions (compatibility)	Nurse		

Oral drug administration	Nurse		
IV drug Administration	Nurse		
Cannula patency check	Nurse	Before IV drug administration	Test if line is patent with water for injection.
Checking cannula sites	Nurse	During shift changes/twice a day	Visual inspection and palpation of the soft tissue for localized infection.
Giving Vitamin K	Nurse	At birth/as required	
Routine cord care - antiseptic application	Nurse/patient's family	Once daily	
Eye care - routine drops application	Nurse/ patient's family	Once daily	
OPV vaccination	Nurse	As required	
BCG vaccination	Nurse	As required	
**Oxygen**			
Checking tube position and nostril care/damage	Nurse	8 times daily/3 hourly	
Initiating and regulating oxygen flow	Nurse	As required	
Documenting oxygen treatment	Nurse/clinician	As required	
Checking and documenting pulse oximetry	Nurse/clinician	3 hourly/as required	For patients on oxygen
Monitoring/regulating pressure	Nurse/clinician	3 hourly/as required	Regulating pressure is done by clinician
Checking nose/cleaning airway	Nurse	3 hourly/as required	
Checking respiration	Nurse	3 hourly/as required	
Checking and changing humidifier	Nurse	As required	
**CPAP management**			
CPAP machine setup	Nurse/clinician	As required	
Applying nasal prongs/fixing tubing	Nurse/clinician	As required	
**Phototherapy**			
Checking eyes for damage	Patient's family under supervision by nurse	4 times daily/6 hourly	
Skin colour	Nurse/family	4 times daily/6 hourly	Conduct together with vital signs
Checking exposure/baby positioning	Shared by clinical team	Continuous/6hourly/per shift	
Fixing eye pad	Patient's family under supervision by nurse	Continuous/6hourly	
Documenting phototherapy	Nurse/clinical team	Shift change/continuous	Done during admissions and as required
**EBM and formula milk preparation**			
Formula making	Nurse/ nutritionist/ mother	8 times daily/3 hourly	
Storage and labelling of expressed breastmilk	Nurse/ nutritionist/ mother	8 times daily/3 hourly	
Measuring volumes for individual patients	Nutritionist	Continuous	
Disinfection of cups	Nurse/patient attendant	8 times daily/3 hourly (after every feed)	
**Feeding**			
Teaching/counselling on breastfeeding (attachment/suck)	Nurse/nutritionist	On admission and as required/daily	
Checking feed prescribed/type of feed	Nurse/mother under supervision	3 hourly or as per feeding schedule	
Cup feeding	Nurse/mother under supervision	8 times daily/3 hourly	

Nasogastric tube feeding/checking nostril	Nurse/mother under supervision	8 times daily/3 hourly	Nostril should be checked by a nurse when administering drugs
Checking residual gastric volumes (nasogastric aspiration)	Nurse/mother under supervision	8 times daily/3 hourly	
Charting feed volumes / times	Nurse/mother under supervision	8 times daily/3 hourly	
Nasogastric tube insertion	Nurse	As required and replace after every 3 days	
**Blood transfusion/ exchange transfusion**			
Cross-checking blood for transfusion with co-worker	Nurse	As required	
Transfusion chart (patient observations/volume of blood)	Nurse	1/4 hourly	
Pre-administration check of laboratory results/medical record	Clinician	As required	
Exchange transfusion progress	Nurse/clinical team	Continuous with clinical team during procedure	
**Documentation**			
Discharge and admission registration	Nurse	As required	Nurses would benefit from clerical assistants, but nurses are responsible.
Patient labels	Nurse	As required	
Notifications – Birth	Nurse	As required	
Notifications – Death	Clinician/HRIO	As required	
Treatment sheets review	Nurse/clinician	Once daily	
Incident book	Nurse	As required	
Updating mother/child health book (vaccines, weight etc.)	Nurse	As required	
Recording in drug books	Nurse	As required	
Billing	Multidisciplinary	As required	Services free in public hospitals, therefore no billing
Recording of Stocks – non-pharmaceuticals	Nurse	As required	
Managing medical records	Nurse/clinical team	Continuous/as required	
**Counselling /support**			
Parent - counselling, answering questions about clinical/nursing care	Nurse	Continuous/as required	
Support for KMC	Shared with clinician and patient's family	As required	
Supervision of mother during KMC	Nurse/clinician	As required	Experienced mothers could assist in helping the other mothers
Expressing breastmilk	Nurse/ nutritionist/ experienced mothers	As required	
Health education and progress	Nurse	As required	
Post-discharge care advice	Nurse	On discharge/as required	
Instructions for drugs/medication on discharge	Nurse/pharmacist	On discharge	Nurse reinforces medication instructions for post-discharge use during the discharge process

			after or before patient obtains medication from the pharmacy, depending on hospital policy.
Family planning	Nurse	As required	
HIV/STI prevention	Nurse/ counsellor/ clinician	As required	
Bereavement counselling	Nurse/clinician	As required	
**Infection control and cleaning**			
Cot cleaning	Support staff	Daily cleaning and thorough cleaning after discharge of a baby before another uses it and as required	
Cleaning incubator	Support staff		
Hand washing	Multidisciplinary	As required	
Visitors education/practice (on gowns/shoes/hand hygiene)	Nurse	As required	
**Miscellaneous**			
Providing input to medical ward rounds	Nurse	During ward rounds	
Accompany to lab/X-ray/theatre for procedure or operation	Nurse	As required	
Accompany on outward referral to another facility	Nurse	As required	
Last offices (stopping interventions and preparing documentation).	Nurse	As required	Washing the body and anything else should be done by mortuary or support staff.
Pre-operative and post-operative care	Nurse	As required	
Assistance with portable chest X-ray	Nurse/radiology team	As required	
Preoperative and postoperative care	Nurse	As required	
Setting alarms (incubator)	Nurse	As required	Part of 6 hourly review and checks
Equipment checks	Nurse/biomedical team	As per schedule/as required	
Equipment handover	Nurse	Once per shift	

Tasks listed as being done by nurses can also be done by students under supervision of a qualified nurse, who is responsible for confirming that the task has been done correctly and as per hospital policy. However, students are not to carry out any tasks for category A patients.

Abbreviations: IV: intravenous, KMC: kangaroo mother care, HRIO: health records and information officer.

Extracted from: Murphy GA V, Omondi GB, Gathara D, et al. Expectations for nursing care in newborn units in Kenya: moving from implicit to explicit standards. BMJ Global Health 2018;3:e000645. doi:10.1136/bmjgh-2017-000645
