# Supplementary material for: Missed nursing care in newborn units: a cross-sectional direct observational study
Source: BMJ Qual Saf. 2019 Jun 6;29(1):19–30. doi: 10.1136/bmjqs-2019-009363 (PMC6923939; doi:10.1136/bmjqs-2019-009363)
Supplement: Supplementary data [file bmjqs-2019-009363supp003.pdf]

Supplementary table 2: Characteristics of babies observed by hospital and sector

		Mission		Private		Public		
		Hospital 1	Hospital 2	Hospital 3	Hospital 4	Hospital 5	Hospital 6	Overall
Shift of observation								
	weekday day	9(25.0)	10(27.8)	10(27.8)	9(25.0)	10(27.8)	11(30.6)	59[27.3]
	weekday night	9(25.0)	9(25.0)	10(27.8)	9(25.0)	9(25.0)	8(22.2)	54[25.0]
	weekend day	9(25.0)	8(22.2)	8(22.2)	9(25.0)	8(22.2)	8(22.2)	50[23.1]
	weekend night	9(25.0)	9(25.0)	8(22.2)	9(25.0)	9(25.0)	9(25.0)	53[24.5]
Child category								
	A(Critical/HDU)	12(33.3)	12(33.3)	11(30.6)	11(30.6)	11(30.6)	12(33.3)	69[31.9]
	B(Acute)	12(33.3)	12(33.3)	13(36.1)	13(36.1)	13(36.1)	12(33.3)	75[34.7]
	C(Stable)	12(33.3)	12(33.3)	12(33.3)	12(33.3)	12(33.3)	12(33.3)	72[33.3]
Gender								
	Male	24(66.7)	23(63.9)	25(69.4)	12(33.3)	18(50.0)	20(57.1)	122[56.7]
	Female	12(33.3)	13(36.1)	11(30.6)	24(66.7)	18(50.0)	15(42.9)	93[43.3]
Age categories (days)								
	<=2 days	9(25.0)	11(30.6)	7(19.4)	6(16.7)	7(19.4)	9(27.3)	49[23.0]
	3 - 7 days	14(38.9)	9(25.0)	13(36.1)	11(30.6)	13(36.1)	20(60.6)	80[37.6]
	8 - 28 days	13(36.1)	16(44.4)	16(44.4)	19(52.8)	16(44.4)	4(12.1)	84[39.4]
Pooled birth weight categories								
	<1.4kg	8(22.2)	5(14.3)	11(30.6)	23(63.9)	10(27.8)	13(36.1)	70[32.6]
	1.5-<1.9	14(38.9)	3(8.6)	11(30.6)	1(2.8)	17(47.2)	4(11.1)	50[23.3]
	2.0-<2.4	1(2.8)	10(28.6)	3(8.3)	3(8.3)	2(5.6)	3(8.3)	22[10.2]
	>=2.5	13(36.1)	17(48.6)	11(30.6)	9(25.0)	7(19.4)	16(44.4)	73[34.0]
Nurse_Patient ratio								
	1 - 3 patients/nurse	0(0.0)	33(91.7)	16(44.4)	35(97.2)	0(0.0)	0(0.0)	84[39.1]
	4 - 7 patients/nurse	14(40.0)	3(8.3)	20(55.6)	1(2.8)	6(16.7)	6(16.7)	50[23.3]
	>=8 patients/nurse	21(60.0)	0(0.0)	0(0.0)	0(0.0)	30(83.3)	30(83.3)	81[37.7]
Type of delivery								
	SVD	21(58.3)	2(5.6)	7(20.6)	5(13.9)	26(72.2)	20(55.6)	81[37.9]
	CS	15(41.7)	32(88.9)	24(70.6)	29(80.6)	10(27.8)	16(44.4)	126[58.9]
	AVD	0(0.0)	2(5.6)	3(8.8)	2(5.6)	0(0.0)	0(0.0)	7[3.3]
Admission/current diagnosis								
	Premature,LBW	21(58.3)	4(11.1)	20(55.6)	14(38.9)	19(52.8)	14(38.9)	92[42.6]
	Respiratory Distress Syndrome	2(5.6)	16(44.4)	2(5.6)	11(30.6)	7(19.4)	4(11.1)	42[19.4]
	Jaundice	1(2.8)	3(8.3)	7(19.4)	3(8.3)	0(0.0)	10(27.8)	24[11.1]
	Birth asphyxia	6(16.7)	3(8.3)	0(0.0)	0(0.0)	5(13.9)	3(8.3)	17[7.9]
	Neonatal sepsis	0(0.0)	5(13.9)	1(2.8)	1(2.8)	1(2.8)	1(2.8)	9[4.2]
	Meconium aspiration,	0(0.0)	2(5.6)	0(0.0)	1(2.8)	1(2.8)	3(8.3)	7[3.2]
	Hypoxic Eschemic Encephalopathy	4(11.1)	0(0.0)	0(0.0)	1(2.8)	0(0.0)	0(0.0)	5[2.3]
	For observation or accommodation	0(0.0)	1(2.8)	6(16.7)	0(0.0)	1(2.8)	0(0.0)	8[3.7]
	Other	2(5.6)	2(5.6)	0(0.0)	5(13.9)	2(5.6)	1(2.8)	12[5.6]
