# Supplementary material for: Missed nursing care in newborn units: a cross-sectional direct observational study
Source: BMJ Qual Saf. 2019 Jun 6;29(1):19–30. doi: 10.1136/bmjqs-2019-009363 (PMC6923939; doi:10.1136/bmjqs-2019-009363)
Supplement: Supplementary data [file bmjqs-2019-009363supp004.pdf]

Supplementary table 3: Number of expected tasks as per neonatal nursing guidelines and the proportion of these tasks completed by hospital sector and neonatal care category

		Hospital sector						Neonatal care category					
		Mission		Private		Public		Critical/HDU		Acute		Stable	
		Expected tasks	Tasks done (%)	Expected tasks	Tasks done (%)	Expected tasks	Tasks done (%)	Expected tasks	Tasks done (%)	Expected tasks	Tasks done(%)	Expected tasks	Tasks done(%)
**General nursing**	Handing over of patient	72	72(100.0)	72	72(100.0)	72	66(91.7)	69	69(100.0)	75	72(96.0)	72	69(95.8)
	Patient assessment done	72	11(15.3)	72	40(55.6)	72	32(44.4)	69	33(47.8)	75	25(33.3)	72	25(34.7)
	Baby cleaned	37	20(54.1)	34	21(61.8)	55	42(76.4)	37	24(64.9)	45	32(71.1)	44	27(61.4)
	Linen changed	37	22(59.5)	34	30(88.2)	55	18(32.7)	37	18(48.6)	45	30(66.7)	44	22(50.0)
	Nurse attends ward round	28	28(100.0)	37	36(97.3)	10	0(0.0)	29	25(86.2)	22	19(86.4)	24	20(83.3)
	Elimination care done	72	71(98.6)	72	68(94.4)	72	68(94.4)	69	66(95.7)	75	72(96.0)	72	69(95.8)
	Communication to parent	72	47(65.3)	72	35(48.6)	72	23(31.9)	69	28(40.6)	75	37(49.3)	72	40(55.6)
	Hand washing/scrub	72	72(100.0)	72	72(100.0)	72	56(77.8)	69	69(100.0)	75	75(100.0)	72	56(77.8)
	Cord care where required	38	22(57.9)	28	8(28.6)	44	12(27.3)	43	17(39.5)	39	15(38.5)	28	10(35.7)
	¥Temperature monitored	72	61(84.7)	72	60(83.3)	72	6(8.3)	69	41(59.4)	75	45(60.0)	72	41(56.9)
	¥Respiration monitored	72	48(66.7)	72	58(80.6)	72	1(1.4)	69	39(56.5)	75	40(53.3)	72	28(38.9)
	¥Pulse monitored	72	56(77.8)	72	62(86.1)	72	4(5.6)	69	42(60.9)	75	44(58.7)	72	36(50.0)
	¥Oxygen saturation monitored	48	9(18.8)	48	36(75.0)	48	4(8.3)	69	24(34.8)	75	25(33.3)	0	0(.)
	Turning done as required	72	23(31.9)	72	33(45.8)	72	25(34.7)	69	27(39.1)	75	30(40.0)	72	24(33.3)
	Feeding 4hourly as required	55	44(80.0)	63	40(63.5)	62	42(67.7)	44	27(61.4)	64	43(67.2)	72	56(77.8)
**Phototherapy care**	Eye care done	6	2(33.3)	13	6(46.2)	15	4(26.7)	11	4(36.4)	15	5(33.3)	8	3(37.5)
	Eye pad changed	6	2(33.3)	13	6(46.2)	15	4(26.7)	11	4(36.4)	15	5(33.3)	8	3(37.5)
	Skin assessment 1	6	3(50.0)	13	7(53.8)	15	9(60.0)	11	5(45.5)	15	10(66.7)	8	4(50.0)
	Skin assessment 2	6	0(0.0)	13	5(38.5)	15	0(0.0)	11	0(0.0)	15	2(13.3)	8	3(37.5)

	Check eyes for damage 1	6	4(66.7)	13	12(92.3)	15	15(100.0)	11	9(81.8)	15	15(100.0)	8	7(87.5)
	Check eyes for damage 2	6	1(16.7)	13	10(76.9)	15	8(53.3)	11	6(54.5)	15	7(46.7)	8	6(75.0)
	Turning/positioning done 1	6	4(66.7)	13	13(100.0)	15	14(93.3)	11	10(90.9)	15	13(86.7)	8	8(100.0)
	Turning/positioning done 2	6	3(50.0)	13	11(84.6)	15	12(80.0)	11	8(72.7)	15	11(73.3)	8	7(87.5)
	Turning/positioning done 3	4	1(25.0)	13	5(38.5)	15	8(53.3)	9	4(44.4)	15	7(46.7)	8	3(37.5)
**Oxygen therapy care**	Oxygen regulated	23	14(60.9)	32	26(81.3)	21	21(100.0)	56	44(78.6)	20	17(85.0)	0	0(.)
	Check nostril tube position 1	23	16(69.6)	31	29(93.5)	21	16(76.2)	56	43(76.8)	19	18(94.7)	0	0(.)
	Check nostril tube position 2	23	12(52.2)	32	27(84.4)	21	3(14.3)	56	25(44.6)	20	17(85.0)	0	0(.)
	Check nostril tube position 3	23	12(52.2)	32	27(84.4)	21	3(14.3)	56	29(51.8)	20	13(65.0)	0	0(.)
	Check nostril tube position 4	23	11(47.8)	32	24(75.0)	21	1(4.8)	56	24(42.9)	20	12(60.0)	0	0(.)
**IV fluids**	Already on IV fluids monitored	8	3(37.5)	4	4(100.0)	9	9(100.0)	15	13(86.7)	5	3(60.0)	1	0(0.0)
**IV treatment**	IV treatment canula flushed	49	25(51.0)	46	24(52.2)	31	2(6.5)	58	22(37.9)	48	15(31.3)	20	14(70.0)
**KMC**	KMC support	12	10(83.3)	5	5(100.0)	15	14(93.3)	1	1(100.0)	4	4(100.0)	27	24(88.9)
	KMC supervision	12	7(58.3)	5	5(100.0)	15	12(80.0)	1	1(100.0)	4	3(75.0)	27	20(74.1)
**Documentation**	Neonatal assessment	72	15(20.8)	72	50(69.4)	72	42(58.3)	69	45(65.2)	75	36(48.0)	72	26(36.1)
	Planned care	72	65(90.3)	72	43(59.7)	72	32(44.4)	69	50(72.5)	75	44(58.7)	72	46(63.9)
	Vital signs	72	70(97.2)	72	70(97.2)	72	14(19.4)	69	53(76.8)	75	52(69.3)	72	49(68.1)
	Treatment	50	49(98.0)	62	61(98.4)	38	36(94.7)	65	64(98.5)	57	54(94.7)	28	28(100.0)
	Ward round recommendations	28	24(85.7)	37	29(78.4)	10	2(20.0)	29	23(79.3)	22	15(68.2)	24	17(70.8)
	Phototherapy documentation	5	4(80.0)	11	11(100.0)	15	4(26.7)	10	6(60.0)	15	7(46.7)	6	6(100.0)
	Feeds	55	42(76.4)	63	58(92.1)	62	37(59.7)	44	37(84.1)	64	50(78.1)	72	50(69.4)
	Oxygen therapy	23	19(82.6)	32	29(90.6)	21	9(42.9)	56	42(75.0)	20	15(75.0)	0	0(.)
	Health talks/parent communications	72	35(48.6)	72	13(18.1)	72	5(6.9)	69	18(26.1)	75	17(22.7)	72	18(25.0)
	Fluids administered	21	19(90.5)	24	21(87.5)	21	20(95.2)	46	44(95.7)	17	15(88.2)	3	1(33.3)

	Turning/positioning	72	10(13.9)	72	48(66.7)	72	1(1.4)	69	24(34.8)	75	19(25.3)	72	16(22.2)
